# Using a double-plicated posterior leaflet as an anchor for mitral valve replacement: a case of mitral annular calcification

**DOI:** 10.1186/1749-8090-8-58

**Published:** 2013-04-01

**Authors:** Shinichi Taguchi, Tatsuru Niibori, Ichiro Hayashi, Hirofumi Kasahara, Ryohei Yozu

**Affiliations:** 1 Department of Cardiovascular Surgery, Keio University Hospital, 35 Shinanomachi, Shinjuku-ku, Tokyo, 160-8582, Japan; 2 Department of Cardiovascular Surgery, National Hospital Organization Saitama Hospital, Saitama, Japan

**Keywords:** Mitral valve replacement, Mitral regurgitation, Mitral annular calcification, Equine pericardium

## Abstract

We present a 62-year-old man with mitral regurgitation whose posterior annulus had severe calcification. Mitral valve replacement was performed by anchoring the cuff on a double-plicated posterior leaflet, and reinforcing with an equine pericardium. The patient is doing well 13 years after surgery with echocardiography showing no problems.

## Background

Mitral regurgitation (MR) with severe mitral annular calcification (MAC) forces surgeons into a difficult situation when deciding on a surgical method. Many authors have described specific mitral valve replacement (MVR) methods for MAC. Although managing a patient to prevent complications in the early postoperative phase is important, long-term stability without recurrence of MR is the bottom line for a patient’s quality of life, even with MAC. We were able to obtain good results 13 years after an atypical valve operation. This report evaluates the operation and the results.

## Case presentation

**Figure 1:**
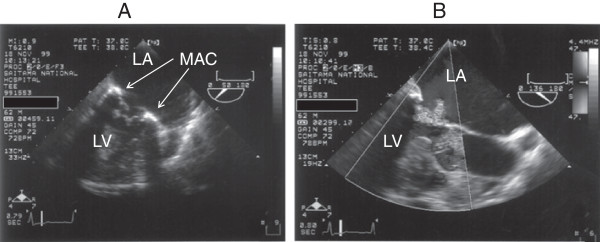
**(A) Preoperative transesophageal echocardiogram revealed serious mitral annular calcification.** (**B**) Doppler echocardiogram revealed severe mitral regurgitation due to chordae rupture of the posterior medial scallop. MAC: mitral annular calcification; LA: left atrium; LV: left ventricle.

A 62-year-old man (height 156.7 cm, weight 43.8 kg, body surface area 1.40 m^2^) presented at our affiliated National Hospital Organization Saitama Hospital in September 1999 with orthopnea after 2 months of dyspnea on exertion and palpitation. He had histories of hypertension and brain infarction. He was hospitalized with a diagnosis of MR and atrial fibrillation. After treating acute heart failure, he was scheduled for surgery in November 1999. Preoperative transesophageal echocardiography revealed severe MR due to chordae rupture of the posterior medial scallop with serious MAC (Figure 1A,B).

### Operative procedure

**Figure 2:**
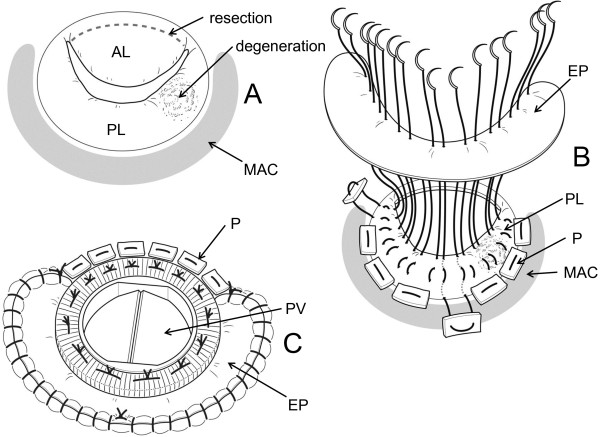
**(A) Degeneration of posterior medial scallop and annular calcification of posterior leaflet were noticed.** Anterior leaflet was resected. (**B**) Posterior leaflet was plicated twice, and the sutures were passed through an equine pericardium. (**C**) The prosthetic valve was fixed with the above-mentioned sutures in the posterior area, and in the conventional everting manner in the anterior area. The equine pericardium was sutured to the left atrial wall to cover the annular calcification. AL: anterior leaflet; PL: posterior leaflet; MAC: mitral annular calcification; EP: equine pericardium; P: pledgets; PV: prosthetic valve.

Operative findings for the mitral valve were degeneration of the posterior medial scallop in the prolapsed area, and severe annular calcification of the posterior leaflet (Figure 2A). MVR was decided due to degeneration and existence of MAC. Anterior leaflet was resected, leaving the posterior leaflet preserved. 2–0 polyester pledgeted mattress sutures were placed on the posterior leaflet itself to plicate it twice. Most of the sutures did not penetrate the MAC, leaving it intact (Figure 2B). The sutures were passed through an equine pericardium, and then through the cuff of a standard 25-mm Carbomedics Mitral Valve (Sulzer Carbomedics Inc., Austin, TX, USA). The equine pericardium was sutured to the left atrial wall (LA) with 4–0 polypropylene running stitches to cover the MAC (Figure 2C). The anterior side of the artificial valve was sutured to the annulus with conventional everting mattress stitches.

**Figure 3:**
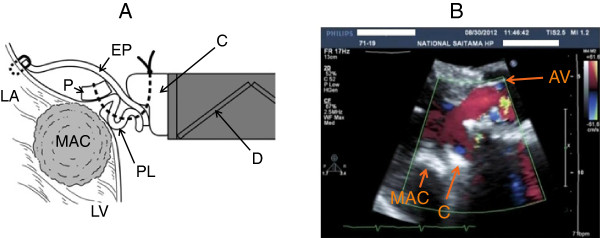
**(A) Cross-sectional view of our operation using a standard prosthetic mitral valve.** The area around the posterior leaflet and the annular calcification is shown. (**B**) The systolic phase in the parasternal long-axis view of the two-dimensional echocardiogram, taken more than 12 years after surgery, shows no perivalvular leakage. AV: aortic valve; C: cuff; D: disc; P: pledget; EP: equine pericardium; PL: posterior leaflet; MAC: mitral annular calcification; LA: left atrial wall; LV: left ventricular wall.

Figure 3A shows a cross-sectional view of the sutured posterior leaflet area. The plicated posterior leaflet and the equine pericardium are placed under and outside the cuff in order not to obstruct closure of the disc.

Post-operative course was without significant problems. Since discharge, he has been followed-up for 13 years. He is enjoying a normal life without major symptoms. A recent transthoracic echocardiogram showed no perivalvular leakage (Figure 3B) and no problems around the pericardial patch.

## Discussion

This is a report on an operative technique for managing MAC, which emphasizes durability, and on long-term follow-up over a period of 13 years. The results of recent echocardiography around the prosthetic valve were excellent, and the patient is doing very well. Few reports of such a long-term follow-up after this type of surgery have been published.

MVR methods for MR with MAC can be divided into 2 types. One is a technique that attempts to resect MAC as much as possible, and the other leaves MAC intact by devising a method for suturing the prosthesis.

Surgery to resect MAC is often challenging because calcification is often deep, and there is a probable risk when excavating the area around the posterior atrio-ventricular groove of rupture, injury to circumflex artery, and thrombo-embolic events [1]. To prevent these events, ultrasonic debridement is often used, and methods such as using autologous or equine pericardium [2], and transferring the anterior leaflet to the posterior area [3] are adopted.

The best way to prevent these disadvantages when resecting MAC is to leave it intact, and to develop new methods of suturing the prosthetic valve. Many reports have been published on possible solutions such as: suturing the prosthesis to the LA itself [1], developing a collar for the prosthesis [4], using the leaflets to secure the prosthesis [5], plicating both mitral leaflets and the LA to create a new annulus [6], and using a Dacron graft between the LA and the prosthesis.

The advantages of our method are that folding the posterior leaflet twice creates a new annulus above the MAC, and using the equine pericardium reinforces the suture and acts as a preventive sheet to avoid perivalvular leakage. By preserving the posterior leaflet, the subvalvular apparatus is also maintained. Our method of suturing the prosthesis to the posterior leaflet is similar to that of Di Stefano et al. [6], who plicated the mitral leaflets and the atrial wall to create a new annulus. Although we used only the equine pericardium-covered posterior leaflet as a new annulus, we plicated the leaflet twice. This made the leaflet more solid and suitable as an anchor for suturing the prosthesis. Equine pericardium was used to reinforce this anchor because the leaflet alone might not be structurally sufficient even if plicated twice.

Feindel et al. [2] used pericardium to reconstruct the MAC resected area and to create a new annulus for MVR. Our usage of pericardium is similar to them in the aspect of being a part of the new annulus for MVR, but different from them in the aspect of constituting the new annulus together with the posterior leaflet.

We must mention that this operation was performed in only 1 patient. Although long-term durability of the procedure was proven in the presented case, multiple experiences using the procedure are needed for further evaluation.

## Conclusions

In summary of the presented case, using the double-plicated posterior leaflet as an anchor in MVR and equine pericardium as a reinforcement resulted in an excellent long-term outcome for MR with MAC.

## Consent

Written informed consent was obtained from the patient for publication of this report and any accompanying images.

## Competing interests

The authors declare that they have no conflicts of interests.

## Authors’ contributions

ST and TN contributed to the pre-operative planning and operation of the case. All authors contributed to following up the case. All authors read and approved the final manuscript.
